# Detection of Potassium Deficiency and Momentary Transpiration Rate Estimation at Early Growth Stages Using Proximal Hyperspectral Imaging and Extreme Gradient Boosting

**DOI:** 10.3390/s21030958

**Published:** 2021-02-01

**Authors:** Shahar Weksler, Offer Rozenstein, Nadav Haish, Menachem Moshelion, Rony Wallach, Eyal Ben-Dor

**Affiliations:** 1 Porter School of Environment and Earth Sciences, Faculty of Exact Sciences, Tel Aviv University, Tel Aviv 6997801, Israel; bendor@tauex.tau.ac.il; 2 Institute of Soil, Water and Environmental Sciences, Agricultural Research Organization, Rishon LeZion 7528809, Israel; offerr@volcani.agri.gov.il; 3 The Robert H Smith Institute of Plant Sciences and Genetics in Agriculture, The Hebrew University of Jerusalem, Rehovot 7610001, Israel; nadav.haish@mail.huji.ac.il (N.H.); menachem.moshelion@mail.huji.ac.il (M.M.); 4 Department of Soil and Water Sciences, The Robert H. Smith Faculty of Agriculture, Food and Environment, The Hebrew University of Jerusalem, Rehovot 76100, Israel; rony.wallach@mail.huji.ac.il

**Keywords:** hyperspectral remote sensing, phenomics, functional phenotyping, transpiration rate, potassium, XGboost, reflectance

## Abstract

Potassium is a macro element in plants that is typically supplied to crops in excess throughout the season to avoid a deficit leading to reduced crop yield. Transpiration rate is a momentary physiological attribute that is indicative of soil water content, the plant’s water requirements, and abiotic stress factors. In this study, two systems were combined to create a hyperspectral–physiological plant database for classification of potassium treatments (low, medium, and high) and estimation of momentary transpiration rate from hyperspectral images. PlantArray 3.0 was used to control fertigation, log ambient conditions, and calculate transpiration rates. In addition, a semi-automated platform carrying a hyperspectral camera was triggered every hour to capture images of a large array of pepper plants. The combined attributes and spectral information on an hourly basis were used to classify plants into their given potassium treatments (average accuracy = 80%) and to estimate transpiration rate (RMSE = 0.025 g/min, R^2^ = 0.75) using the advanced ensemble learning algorithm XGBoost (extreme gradient boosting algorithm). Although potassium has no direct spectral absorption features, the classification results demonstrated the ability to label plants according to potassium treatments based on a remotely measured hyperspectral signal. The ability to estimate transpiration rates for different potassium applications using spectral information can aid in irrigation management and crop yield optimization. These combined results are important for decision-making during the growing season, and particularly at the early stages when potassium levels can still be corrected to prevent yield loss.

## 1. Introduction

Plant transpiration rate (TR) is an important trait that reflects the plant water influx and outflux [1,2]. When water is not a limiting factor, the TR depends on abiotic factors such as soil salinity and water vapor in the atmosphere, as well as photosynthetically active radiation (PAR). On the other hand, when water is a limiting factor, there will be a decline in TR once the volumetric water content in the pot or soil reaches a critical level [3]. A decline in TR, or plant water status, may affect plant growth, productivity, and crop quality [4,5]. The ability to measure plant TR through contact or proximal measures can aid in irrigation management and crop-yield optimization [6]. This is because TR will decline with the soil water content, which may be used as a decision aid for irrigation before the plant water stress results in tissue damage and yield loss [7]. Depending on the plant growth stage and phenological cycle, such measurements and appropriate decisions must be made rapidly and accurately. Leaf gas exchange, including TR, is usually measured manually in a leaf gas-exchange chamber [8]. While such a measurement may be rapid, it is still biased because a single or a few leaves are taken to represent the entire plant, and sometimes an entire field. Measuring multiple leaves on multiple plants may take several hours, during which time changes in ambient conditions such as PAR and humidity may skew the results. 

Accordingly, a rapid solution for estimating TR in multiple leaves and whole plants is desirable. One solution is the process of collecting a large amount of data focused on form and function, termed high-throughput phenotyping [9]. The analysis of these data to understand variations between different plant genomes is termed phenomics [10]. Phenomics has been recognized as a useful tool for sustaining agricultural development along with the advances in gene discovery and trait expression [11]. Attempts to maintain food security and support food production are manifested in the rapid development of phenomics facilities. Such facilities are usually greenhouses with automated irrigation systems and specialized photography chambers to capture images; the latter are analyzed to support the breeding and cultivation chain or to preselect the best plants for transplantation in the field [12]. The potential for rapid, early screening of “bad” plants may help breeding procedures in allowing the selection of lines with desirable traits to be used as parental lines in breeding programs. Another solution for estimating TR is satellite remote sensing, which can typically estimate large-scale evapotranspiration [13]. Using a combination of broad-range bands in the visible and near-infrared (NIR) regions, different vegetation indices are calculated and correlated with physiological processes that depend on solar radiation absorbance and reflectance by the plant canopy [14]. However, this technique is limited and cannot estimate the TR itself when it is affected by environmental factors [15].

Greenhouse proximal hyperspectral imaging spectroscopy of vegetation is a relatively new field of study which addresses this difficulty. This imaging technology fuses the image domain with the high-resolution spectral domain, in a nondestructive method that has proven to be a reliable technique in agricultural and agronomic research, from the laboratory to field scale. Recently, it has been introduced into greenhouse scenarios for in-vivo analyses [16,17]. Hyperspectral imaging spectroscopy is usually interpreted and analyzed using one of two methods: reducing the measured signal to a few important bands or considering the entire shape of the spectral curve. The first is often performed using a narrow-spectral-band vegetation index, whereas the latter approach can involve reducing the dimensionality of the spectrum, by partial least squares algorithm [18], or machine-learning and deep-learning approaches such as random forest [19], support vector machine (SVM) [5], or deep neural networks [20].

Extreme Gradient Boosting (XGBoost) algorithm [21] has recently gained popularity as a winning solution in machine-learning competitions. However, the algorithm is relatively new and has been applied to only a few published remote-sensing studies. Georganos et al. [22] used XGBoost to classify WorldView-3 multispectral urban terrain images. Zhong et al. [20] compared XGBoost with other machine-learning algorithms and reported it to be superior in classifying multispectral remote-sensing time-series data of mixed crops. Those authors also compared XGBoost classification results with two types of deep-learning models and reported superior or nearly equal results. Abdi [23] classified multitemporal multispectral land-cover and land-use satellite images of boreal areas using four machine-learning algorithms: XGBoost and SVM were ranked best. Sandino [24] used a hyperspectral camera mounted on an unmanned aerial vehicle to distinguish trees that were deteriorating due to fungal infections from healthy trees and reported an overall accuracy of 97%. Loggenberg et al. [25] used a hyperspectral camera mounted on a tripod in a vineyard to classify water-stressed Shiraz vines with good overall accuracy (80%). While all of these studies used XGBoost as a classifier, none tried to estimate continuous values using XGBoost regression trees.

Traditionally, spectral leaf measurements are carried out using a point spectrometer and a special apparatus in the laboratory or the field and then matched with laboriously collected physiological data to estimate certain vegetative traits such as pigment concentration, water content, or TR [26,27]. The use of an imaging chamber in a greenhouse is faster, although it creates a queue of plants, each being imaged at a different time and affected by changing ambient conditions. Different ambient conditions have already been proven to affect the usefulness of hyperspectral image analysis [28]. When phenomics systems are used to study plant function through physiological trait measurements, they are termed functional phenomics systems [7,9]. Recently, a new high-throughput, whole-plant functional phenotyping system named PlantArray (PA) was developed, which can calculate the momentary and daily TR of an entire array of plants simultaneously, as well as other plant and soil attributes and interactions [29]. The PA system’s dynamic responses to changes in ambient conditions [3], nutrient stress, and biostimulants effectiveness have been recently explored [1].

Potassium is considered an essential plant nutrient for crops. It is involved in many processes and metabolic pathways, such as osmoregulation, enzyme activation, stomatal opening, photosynthesis and stress resistance [30]. Potassium fertilizing increases the plants’ tolerance to water shortage, while a deficit may affect stomatal closure and lead to a higher transpiration rate [30]. The ability to monitor potassium levels in order to prevent yield loss is important and has been examined in several studies. Pandey et al. [17] used a hyperspectral camera and a conveyer belt for the plants to quantitatively estimate chemical properties (such as potassium concentration) of maize and soybean plants. Zhang et al. [31] studied oilseed rape leaves, picked, and brought to a hyperspectral scanning camera to estimate potassium content. Pimstein et al. [18] explored the potential for potassium estimation in field-grown wheat plants using a field spectrometer. A similar investigation was carried out by Mahajan et al. [16]. The latter tried to find the best narrow-band vegetation index to estimate the quantity or concentration of potassium. While the ability to estimate chemical properties was exhibited, destructive methods were required to produce the database used in those analyses.

In this manuscript, we hypothesized that plants treated with low levels of potassium (compared to medium and high levels) might be accurately classified using XGBoost and images from a hyperspectral moving platform in the greenhouse. The results of the XGBoost classification are compared with the traditional support vector machine classifier (SVM). Moreover, we aimed to estimate the momentary TR using the same ensemble learning XGBoost algorithm. The combined ability to classify the administered treatment and estimate TR was tested during the early growth stage of greenhouse-grown pepper (*Capsicum annuum*) plants treated with different potassium and salinity levels.

## 2. Materials and Methods

### 2.1. Sample Preparation and Experimental Setup

Pepper seedlings (*n* = 144), approximately four weeks post-germination, were planted in 3.9-L pots filled with sand. The plants were grown during April–May 2019 in a semi-commercial greenhouse located at the Robert H. Smith Faculty of Agriculture, Food and Environment in Rehovot, Israel. The temperature and relative humidity (RH) in the greenhouse were continuously monitored by the PA meteorological station (Plant-Ditech Ltd., Israel). The temperature was maintained under 35 °C by blowing air through a moist mattress; it ranged between 20 and 35 °C and 26% and 90% RH, respectively, with low values during the night.

### 2.2. Phenotyping Platforms

Plant fertigation (quantity and schedule) was controlled using the PA 3.0 platform (www.plant-ditech.com). The high-throughput functional phenotyping platform was also used to measure the plant’s dynamic status through highly sensitive temperature-compensated load cells used as weighing lysimeters. Each plant was monitored and controlled separately, enabling high precision (Halperin et al., 2017).

**Figure 1:**
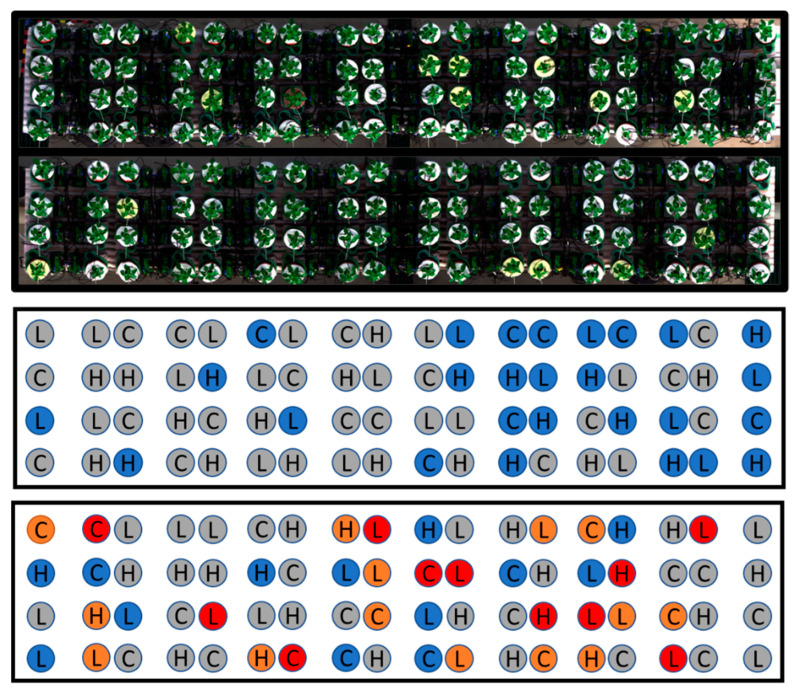
Top: RGB image of the plants, as seen from the hyperspectral camera. Bottom: randomly distributed treatment array on the two experimental tables; nine different treatments were administered using the PlantArray 3.0 system. L: potassium deficit 30 PPM, C: control potassium 105 PPM, H: potassium surplus 180 PPM. Blue cell: H_2_O, orange cell: 0.03 M NaCl, red cell: 0.05 M NaCl, gray cell: removed plants due to root fungi.

The experiment lasted 13 days. The 144 plants were placed on two separate tables (72 plants per table). The plants on the first table were randomly administered three levels of potassium: low, medium, and high, corresponding to 30 PPM, 105 PPM, and 180 PPM, respectively. The plants on the second table were administered three levels of potassium (low, medium, and high) in combination with control, medium, and high levels of salinity (H_2_O, 0.03 M NaCl, and 0.05 M NaCl, respectively) for a total of nine treatments (Figure 1). At the end of the growing period, the plants were extracted from the pots and examined; 45 plants from the first table and 34 plants from the second table were infected with varying root fungi levels. To minimize the effect of disease on the analysis, the infected samples, as judged by a strict criterion, were eliminated; even plants with a low infection level were removed from the study. The remaining number of samples was sufficient for model training and testing.

**Figure 2:**
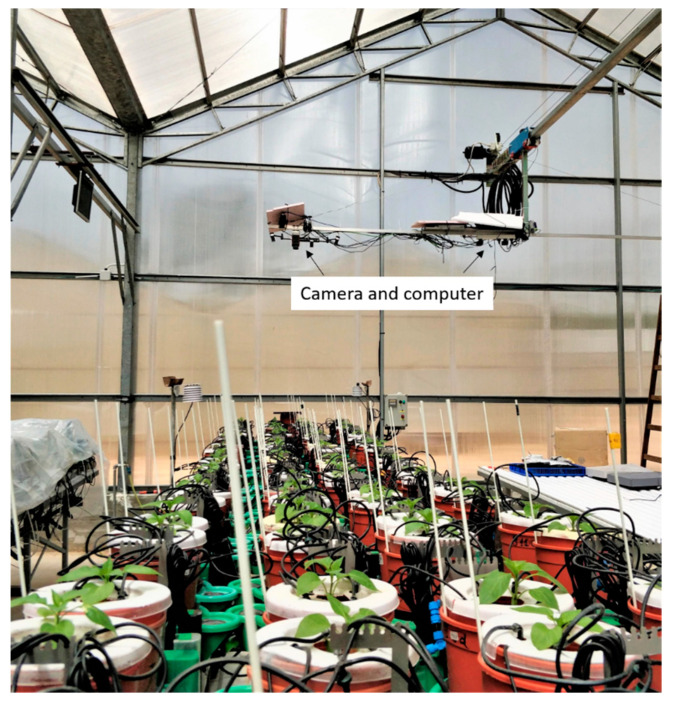
A view of the experimental table and the hyperspectral camera traveling above. Each plant was placed in a custom weighing lysimeter connected to Plantarray 3.0 system. Four drippers irrigated each plant while the water evaporation was restricted using a plastic cap.

The pots were placed into a PA-specific drainage container, situated on a lysimeter (Figure 2). A rubber ring was used to establish a perfect fit between the pot and the container to prevent evaporation. The pot was covered with a custom-designed cover with a hole in its center for the plant stem to emerge and four additional holes for dripper irrigation, which aided in the uniform spread of irrigation. Irrigation was administered during the night, and water drainage was collected at the bottom of the plastic vessel to maintain pot capacity during the day. Weight loss during the daytime was logged and used to calculate the momentary TR. The momentary TR was calculated following Halperin et al. (2017) and adjusted as in Weksler et al. (2020). The difference in plant weight at time *t_i_* and *t*_i−1_ was measured every 3 min. The weight loss was attributed to water leaving the plant through the stomata and the TR was defined by the first derivative of logged weight readings throughout the day.

### 2.3. Image Acquisition and Database Building

Hyperspectral images were acquired every hour during the day (07:00–17:00 h) using a custom-made semi-autonomous linear platform built on the greenhouse roof (Weksler et al., 2020). The greenhouse panels are made of a clear PVC material that diffuses the incoming solar radiation. The platform carried a push-broom hyperspectral radiometrically calibrated camera (FX10, Specim, Finland) with a 400–1000 nm spectral range divided to 448 spectral channels and 1024 pixels in a line, with a field of view of 38° (Figure 2). A small laptop and a microcontroller controlled the camera. The camera’s height and focus were adjusted during the experiment to account for the plant growth. Every image scene included the entire array of plants on both tables.

**Figure 3:**
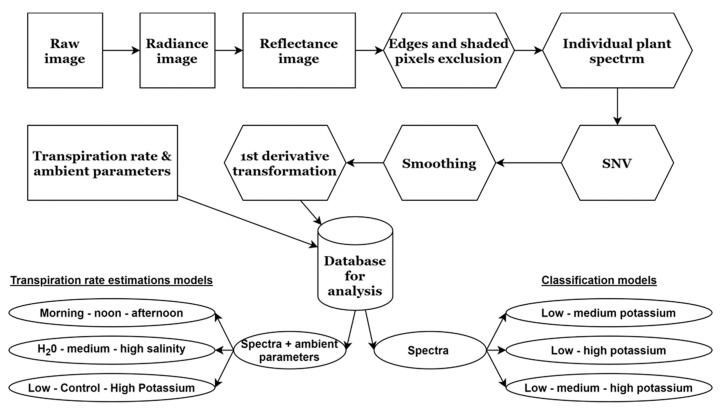
Steps in the processing of every image into an individual plant spectrum, resulting in the full database for analysis and the different models that were tested.

Following image acquisition, the raw images were calibrated to radiance using a pre-calculated gain image calibrated by the manufacturer and an offset image acquired by a closed-shutter image before every image was taken (see Weksler et al., 2020 for the exact calibration equation). Then reflectance was calculated using a reflectance panel (99% reflectance, Spectralon, Labsphere Inc., North Sutton, USA) placed in the scene, which was also used to calibrate the exposure time before each overpass to prevent saturation across all the bands. To generate the plant spectral database, each plant’s mean reflectance signal was then extracted, and shaded pixels were excluded by selecting the brightest 80% of the pixels as a threshold. In addition, mixed pixels (leaf edges) were also excluded using an Otsu filter [32]. Therefore, each plant’s mean reflectance was the mean of hundreds of pixels of leaves in different viewing angles. The edges of the sensor’s spectral range were trimmed, and the subsequent analysis was performed for 425–990 nm with 416 spectral bands. Each plant’s spectral signature was standardized to unit vector by removing the mean and dividing by the standard deviation (SNV transformation), followed by a first-derivative transform and a Savitzky-Golay smoothing transformation with a window size of 7 and a second-polynomial degree (Figure 3). After 13 days, when the plants’ canopies overlapped, the ability to separate individual plants was greatly reduced, and image acquisition was stopped. Several times during the experiment, technical malfunctions prevented camera operation, which reduced the size of the database relative to its full potential. The spectral database was merged with the TR loggings to create the database for the analyses. The final database consisted of 7617 spectral and TR measurements.

### 2.4. Classifying Potassium

The medium- and high-salinity treatments were not used in the classifier training (Table 1). These treatments were omitted in order to focus on classifying potassium-level differences and not differences caused by interactions with the salinity treatments. 

Three different models were trained to achieve good classification results for the low-potassium treatment, to classify the samples into their given potassium treatment. The different models were: low–high classification, low–medium classification, and low–medium–high classification. By dividing the data as such, we ensure that each model received a balanced dataset and that the three class model can describe a more heterogeneous scenario, such as a field scenario, where different field condition (slope, aggregates, organic matter, and minerology) may cause variation in potassium across the field. All the models were trained and optimized with an emphasis on the ability to classify low levels of potassium. The database was partitioned into the desired classes (Table 2); subsequently, the data were randomly divided into training (70%) and validation (30%) sets for every model. The classification models were trained using each training sample’s spectral reflectance signal as the independent variable (also termed features) and the treatment label as the dependent variable. For each model’s estimation, a normalized confusion matrix was plotted. The confusion matrix illustrates the model’s outcome as actual values and estimated values for each group, facilitating the interpretation of the model’s performance. The model’s performance in separating the plants according to the administered treatment was evaluated by calculating the estimation accuracy, which ranged between 0 and 100%, as

(1) $$\mathrm{Accuracy}=\frac{True\;positive+True\;Negative}{n}$$

where *n* is the number of samples in the estimation. However, since the number of samples differed between the groups, accuracy may be a biased measure. Therefore, additional metrics, termed precision, and the sensitivity matrices were calculated for each model. A model’s sensitivity is defined as the proportion of actual positives (Equation (2)), i.e., a sensitivity value of 100% means that all of the low-potassium plants were correctly classified. A model’s precision is defined as its usefulness, and the proportion of correctly identified samples out of all samples estimated to have that same label (Equation (3)), i.e., a precision value of 100% means that the model correctly labeled all plants that were classified as low-potassium plants. These metrics also ranged between 0 and 100% and were calculated as

(2) $$\mathrm{Sensitivity}=\frac{True\;Positive}{True\;Positive+False\;Negative}$$

(3) $$\mathrm{Precision}=\frac{True\;Positive}{True\;positive+False\;Positive}$$

In estimation, feature importance is a measure of the importance of a feature (band) to the model’s estimation and its physical explanation (“spectral assignment”). A common feature importance metric is the gain, which is defined as the improvement in the score (sensitivity) after a feature is used to add a new split to a tree. The feature importance was calculated as the total gain that the feature contributed to the model. To investigate if the same results may be achieved by reducing the dimensionality of the spectral resolution, the 10 most contributing features (bands) from each classification model were selected. Additional classification analysis was calculated based on those features to compare the different models for further analysis.

Furthermore, classification models were also trained using the support vector machine classifier (SVM) using the same features and training samples to investigate if XGBoost has an advantage over a traditional classifier.

### 2.5. Transpiration Rate Estimation

The database was used to train several models using XGBoost and evaluate their performance. Momentary TR was first estimated by training two different XGBoost models: one using the measured spectra and another with ambient conditions during image acquisition added to the spectra as additional independent variables (features). These parameters (RH, PAR, vapor-pressure deficit, and temperature) were logged using the PA greenhouse weather stations. TR was used as the dependent variable. Since the addition of ambient conditions features was found to produce a better model, the subsequent models were trained using the same features (Figure 3).

To analyze the contribution and interaction of the different periods of the day (morning, noon, afternoon) on model training and TR estimation the database was partitioned into three temporal ranges: morning (0700–1000 h), noon (1100–1300 h), and afternoon (1400–1700 h). In order to compare models between these temporal ranges, the data contained in each of those time intervals were randomly divided for training and testing as previously described, and a new model was trained and tested for each interval.

In addition, to test the effects of the different salinity treatments (H_2_O, medium, and high salinity) on the ability to estimate TR, an independent and new model was trained using only the data from the experimental table that received salinity treatments (*n* = 4311). To validate this model, 300 samples were left out from each salinity treatment (H_2_O, medium, high salinity). The left-out data for each salinity treatment (*n* = 300) were composed of equal portions of potassium treatments—100 samples from each treatment: low, medium, high potassium treatments for each salinity treatment. A total of 900 samples were left out for the model validation. A general model was trained on the remaining training samples (*n* = 3411), and estimations were validated three times.

Furthermore, to test the effects of the different potassium treatments (low, control, and high potassium) on the ability to estimate TR, a new and independent model was trained using the different potassium treatment from the table that did not receive salinity treatments (*n* = 3199). To validate this model, 300 samples were left out from each treatment (low, control, and high potassium) for a total of 900 left out samples. A general model was trained on the remaining training samples (*n* = 2299), and estimations were evaluated three times.

Two measures of model performance were calculated: the root mean squared error (*RMSE*) and Willmott’s index of agreement (*d_r_*) [33]):

(4) $$RMSE=\sqrt{\frac{\sum_{i=1}^{N}{\left(P_{i}-O_{i}\right)}^{2}}{N}}$$

(5) $$d_{r}=\{\begin{array}{cc} 1-\frac{\sum_{i=1}^{N}\left|P_{i}-O_{i}\right|}{2\sum_{i=1}^{n}\left|O_{i}-\bar{O}\right|} & if\overset{N}{\underset{i=1}{\sum }}\left|P_{i}-O_{i}\right|\leq 2\overset{N}{\underset{i=1}{\sum }}\left|O_{i}-\bar{O}\right|\\ \frac{2\sum_{i=1}^{N}\left|O_{i}-\bar{O}\right|}{\sum_{i=1}^{N}\left|P_{i}-O_{i}\right|}-1 & if\overset{N}{\underset{i=1}{\sum }}\left|P_{i}-O_{i}\right|>2\overset{N}{\underset{i=1}{\sum }}\left|O_{i}-\bar{O}\right| \end{array}$$

where *P_i_* and *O_i_* are the predicted and the observed TR, respectively. A lower *RMSE* (closest to 0) and a higher *d_r_* (closest to 1) are desired.

### 2.6. Data Analysis and Algorithm

XGBoost is a supervised ensemble learning approach based on decision trees, utilizing a gradient-boosting technique [21]. The algorithm can be used for either regression or classification and has tunable hyperparameters that significantly affect its accuracy. A weak classifier or regressor tree are used to constantly improve the score and reduce the error. After each iteration, the algorithm predicts the class or attribute for each sample. Samples incorrectly classified receive a higher weight in the next iteration, which forces the algorithm to improve their score in the next iteration. This is principally carried out by defining the objective function (loss function). Unlike other machine-learning algorithms, the objective function holds another term which is the regularization term, which may calculate two regularization parameters (L1, L2) during training to reduce overfitting effects. The hyperparameters are typically trained using a model performance-optimization scheme known as a grid-search technique. Optimization of the model’s hyperparameters was carried out on the training samples to generate the best results from every model. A grid search with 5-fold cross-validation was used to evaluate the best hyperparameters for this experiment. These were the number of trees, the tree depth, the learning rate, regularization parameters, and the percent of features for each tree (Table 2). As with XGBoost, the SVM hyperparameters were tuned, with linear log space intervals for each different classification model. SVM with a radial basis function kernel was shown to be superior for hyperspectral reflectance classification [34,35]; therefore, to produce the best possible accuracy, the parameters C (penalty) and *γ* (kernel scale) were searched. For classification, the metric for optimization was the model’s sensitivity, whereas, for the estimation of TR by regression, these were the RMSE and d_r_.

The interquartile range is the difference between the values below 75% and above 25% of the samples after the data are sorted. TR values greater than a 1.5 interquartile range were considered outliers (*n* = 81) and removed, along with the corresponding spectra. These outliers are most likely the result of momentary interference by greenhouse personnel. The TR data were measured and logged using the PA 3.0 system. Statistical analysis, algorithms, and estimation were performed using Python 3.7 [36].

## 3. Results

### 3.1. Classification Analysis

The ability to train different models for the classification tasks resulted in slightly different hyperparameter combinations for each model. All of the models required 800 or 1000 trees, and the trees were considered deep (8 and 12). A deeper tree will have more splits and will include more of the trained data. The regularization terms were small, and the percent of features selected for each tree was usually low (0.5%). Table 2 summarizes the results of the hyperparameter-tuning procedure.

**Figure 4:**
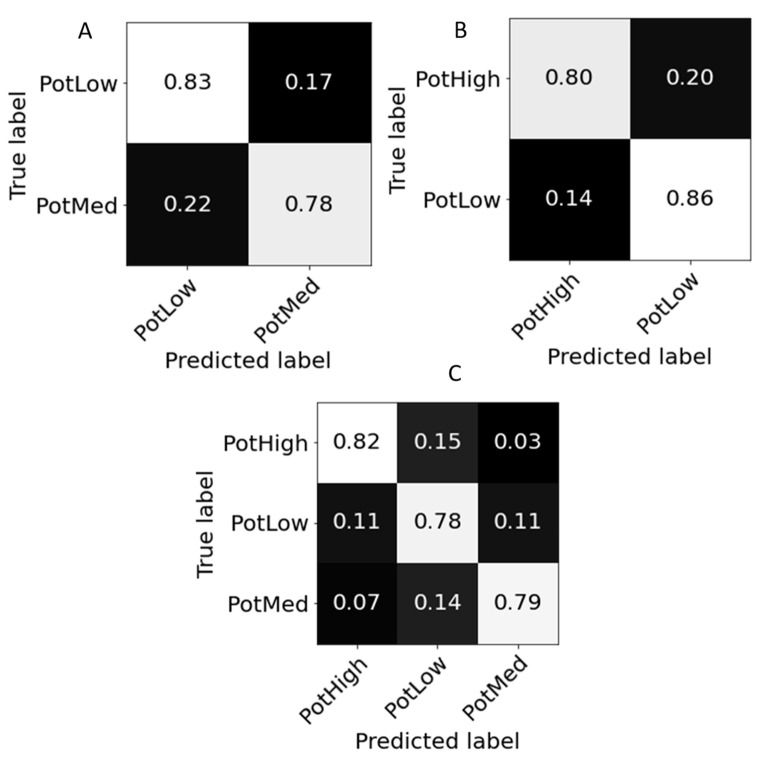
Normalized confusion matrices of XGBoost classifier model of low and medium fertilization treatments (**A**), low and high treatments (**B**), and low, medium, and high treatments (**C**).

The accuracy of each model is presented in Table 3 and shows an overall good ability to classify plants that suffer from low levels of potassium. Three different confusion matrices were plotted (Figure 4). The confusion matrices were normalized for easy comparison between the different models. Separation of low and medium potassium levels showed an overall accuracy of 80% (Table 3), as 83% of the low-potassium samples were correctly classified (Figure 4A). Good precision and sensitivity (81% and 83%, respectively) were also calculated for low potassium treatment. Separation of low and high potassium treatments was better, with an overall accuracy of 83%, as 86% of the low-potassium samples were correctly classified, and good precision and sensitivity were calculated (80% and 86%, respectively). However, the total accuracy for separating low-potassium plants from medium- and high-potassium plants was 79%. While the total accuracy was lower than the former models, 78% of the low-treatment samples were correctly classified, as opposed to 79% and 82% of the medium and high treatments, respectively. Interestingly, the high-treatment group had the best sensitivity and precision (81% and 83%, respectively) as compared to the low-treatment group (76% and 73%, respectively) and medium-treatment group (80% and 81%, respectively). In comparison, the accuracy achieved by the optimized SVM algorithm was much lower for the three different scenarios with 0.67, 0.72, and 0.61 for low-medium, low-high, and low–medium–high models, respectively, whereas XGBoost’s accuracy was 0.80, 0.83, and 0.79, respectively.

**Figure 5:**
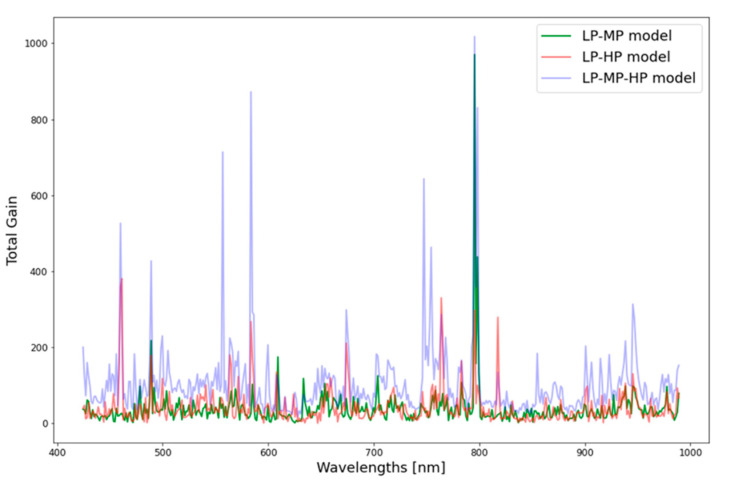
Feature importance in estimation, calculated as total gain a band contributed to the model’s classification of low- and medium-potassium treatments (LP-MP), low and high treatments (LP-HP, and all treatments (LP-MP-HP).

**Figure 6:**
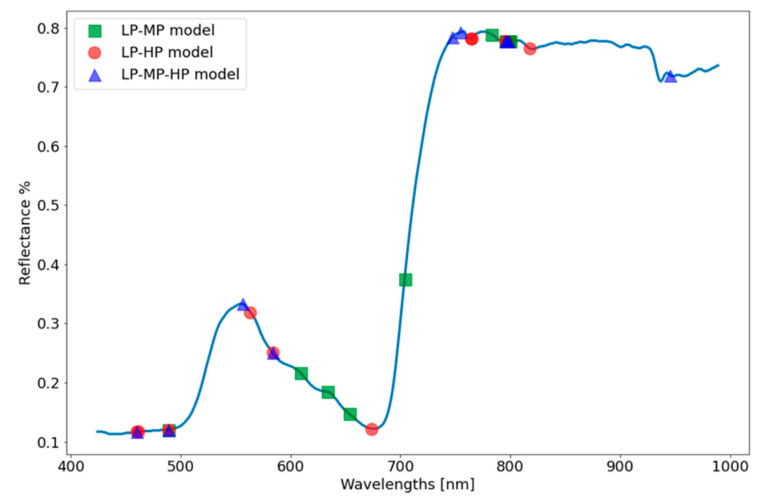
Most important features (bands) in estimating low-potassium plants plotted with a sampled plant spectrum. The 10 most important features are plotted for the low–medium estimation model (red circle), low–high estimation model (green square), and low–medium–high estimation model (blue triangle). Numbers next to a marker indicate the number of models it was found as important in estimation.

The feature importance in the estimation plot is presented in Figure 5. Visual inspection of the plot shows 5–10 bands with higher total gain in the estimation models and many more that have medium or low weights. The 10 best features from each model were joined in a single plot to inspect variation and similarities in the models’ best features (Figure 6). In the low–medium model, most contributing bands were split into three main parts in the blue and green regions, as well as in the NIR plateau and one band in the red-edge. The low–high model was also mostly influenced by bands in the blue region but also by bands in the green and red regions, water-absorption region (~950 nm), and overlapping region with the other models (~800 nm). The three-treatment model was mainly influenced by the blue and red region, NIR plateau overlapping with the two other models, and water-absorption region. Aggregating the features (Figure 6) showed that several bands appear more than once, meaning that they were explicitly found to be important in the different models. The models point to specific regions and several narrow bands that hold most of the spectral information considered important for this treatment classification task. To test this assumption, these best bands (*n* = 22) were combined into a new dataset, and new models for each of the three scenarios were trained using this dataset. The results for each model were worse, and reduced accuracy compared to models based on the full spectral dataset (Table 3).

### 3.2. Estimation of TR

**Figure 7:**
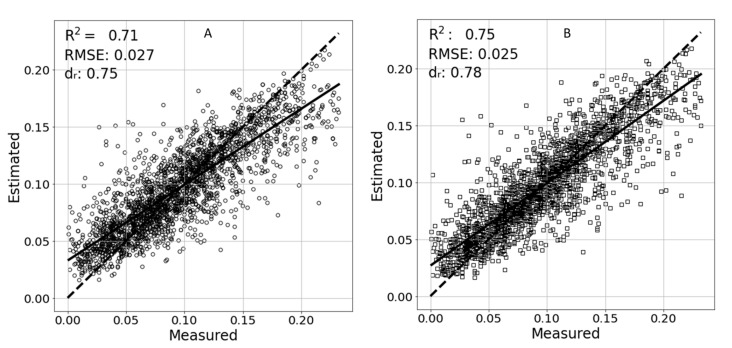
Estimated vs. measured results of the XGBoost model using only the spectra (**A**) and the spectra, including the entire experiment’s ambient features (**B**). A linear trend line of the points is marked with a solid line. An ideal 1:1 result is marked with a dashed line.

Several tests were performed to evaluate the effectiveness of XGBoost to estimate TR from hyperspectral reflectance. First, the data across the entire experiment were used. Using this dataset, with the spectra as the features, a model was trained and estimated an overall error (RSME = 0.027 g/min, an R^2^ = 0.71, and d_r_ = 0.75. The addition of the ambient conditions to the independent features resulted in an improved model with an R^2^ = 0.75 an RMSE = 0.025 g/min and d_r_ = 0.78. The estimation results are plotted in Figure 7.

**Figure 8:**
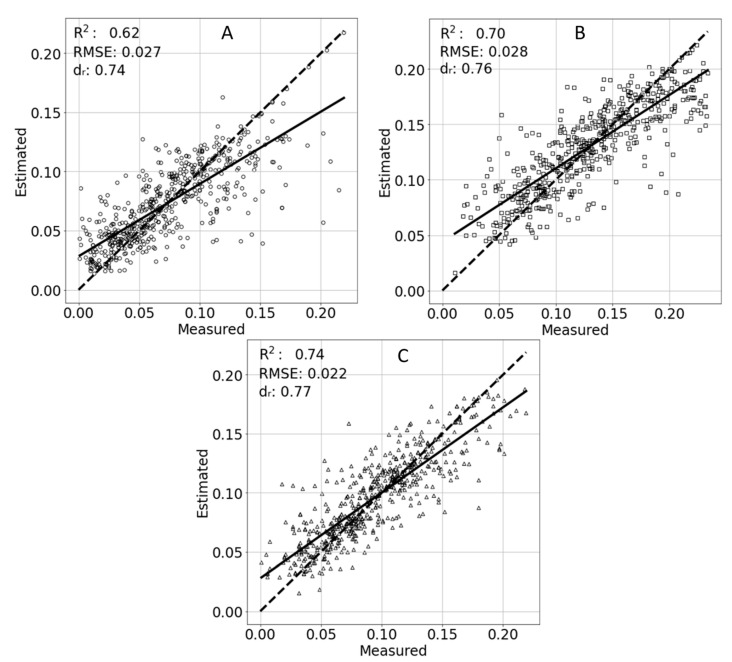
Estimated and measured transpiration rates for the morning (**A**), noon (**B**), and afternoon (**C**). A linear trend line of the points is marked with a solid line. An ideal 1:1 result is marked with a dashed line.

As estimating TR using the ambient features proved superior, those features were included in the subsequently generated models. To compare the different models calculated for each period of the day, the number of samples in each period was randomly down-sampled by the lowest number of samples (*n* = 1970). During the morning, the model resulted in a RMSE = 0.027 g/min with R^2^ = 0.62 and d_r_ = 0.74. A slightly better model fit was achieved at noon with R^2^ = 0.70, with the RSME = 0.028 g/min and d_r_ = 0.76. The best model was achieved in the afternoon, with RMSE = 0.022 g/min, an R^2^ = 0.74 and d_r_ = 0.77 (Figure 8).

**Figure 9:**
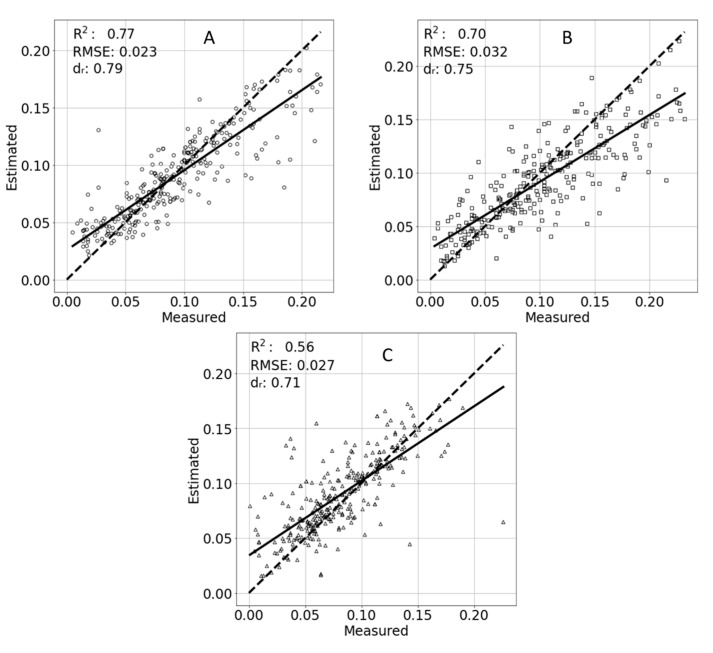
Estimated and measured transpiration rates of different salinity treatments. Water (**A**), medium salinity (**B**), and high salinity (**C**). A linear trend line of the points is marked with a solid line. An ideal 1:1 result is marked with a dashed line.

The results of TR estimation concerning salinity treatments are presented in Figure 9. As salinity increased in a given treatment, a reverse trend in R^2^ was shown. However, the RMSE did not show the same trend. H_2_O treatment resulted in the best estimations of TR with R^2^ = 0.77, RMSE = 0.023 and d_r_ = 0.79. The medium-salinity treatment resulted in R^2^ = 0.71, RMSE = 0.031 and d_r_ = 0.75, and the high-salinity treatment resulted in R^2^ = 0.57, RMSE = 0.027 and d_r_ = 0.71.

**Figure 10:**
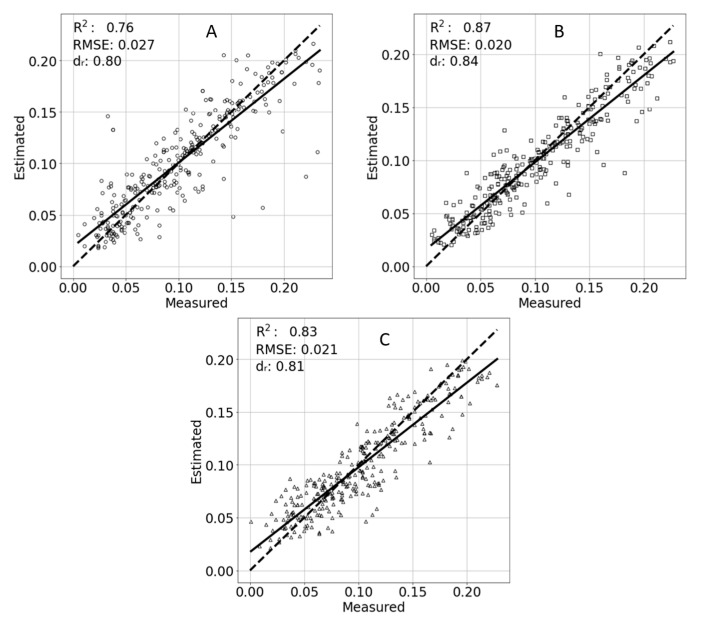
Estimated and measured transpiration rates of different potassium treatments. Low potassium (**A**), control (**B**), and high potassium (**C**). A linear trend line of the points is marked with a solid line. An ideal 1:1 result is marked with a dashed line.

The final model developed was concerned with potassium effects on the estimation of TR and is presented in Figure 10. While the differences in the RMSE are small between the estimations, the medium potassium treatment TR was best estimated with an R^2^ = 0.87, RMSE = 0.02, and d_r_ = 0.84. The high potassium treatment TR was estimated with an R^2^ = 0.83, RMSE = 0.021 and d_r_ = 0.81. Finally the low potassium treatment TR was estimated with an R^2^ = 0.76, RMSE = 0.027 and d_r_ = 0.80.

## 4. Discussion

Remote sensing is often used to analyze nitrogen and pigment content since nitrogen is found in proteins, free amino acids, and chlorophyll. Nitrogen bonds with other elements, resulting in a distinct absorption spectrum and spectral changes in the visible, NIR, and shortwave-infrared portions of the electromagnetic spectrum (usually termed chromophores). The same can be attributed to other elements such as phosphorus and sulfur. However, potassium is found in the plant as a positive ion (K^+^) that does not produce chemical bonds; therefore, it does not produce any absorption feature. While its absence can have distinct effects on plant tissues, in this experiment, the lowest amount of potassium was selected to inhibit biological pathways without causing chlorosis, which can easily be captured by an ordinary camera. Nonetheless, potassium can affect chromophore properties and, as a result, be indirectly monitored via spectroscopy.

Long-term potassium deficiency in leaves usually induces yellowing of the leaves’ margins, i.e., chlorosis [37]. However, insufficient administration of potassium may inhibit or slow biological and physiological pathways without causing leaf chlorosis. Therefore, there are no visible effects on the leaves, and a typical visual inspection or RGB image may not pick up this nutrient deficiency. Nevertheless, the ability to develop models to classify plants that have low potassium levels from those that have received normal and high levels was achieved, owing to the high spectral, spatial and temporal resolution of the images captured during the experiment, along with the supporting attributes logged by the PA system. Unlike other studies in which a single image is analyzed, a large imagery dataset was created and analyzed in this study. As potassium causes no direct alteration to plant reflectance, creating a sizeable spectral dataset facilitated the data analysis and use of the XGBoost algorithm. Typically, spectral reflectance is captured a few times during growing experiments. In this experiment, the high temporal resolution of the hyperspectral images enabled creating a very big dataset. This dataset was best mined using advanced machine learning, which requires thousands of samples to produce accurate and reliable results. Moreover, the high temporal resolution added to the models’ robustness, as they were related to many consecutive days and thousands of spectral and TR measurements, rather than a single measuring event.

Although K^+^ has no direct chromophore, the spectral bands that most contributed to the XGBoost algorithm were, in fact, reflectance bands known to be related to chlorophyll and carotenoid absorption (460 nm and 660 nm) [38,39], light-use efficiency (480–500 and 570–600 nm) [40], red edge (700–720 nm) [41], light scattering, and the leaf-thickness NIR plateau [39]. While only one water-related band at 930–990 nm [42] was found to be one of the most contributing bands, it did influence the model Estimations (Figure 5). This is not surprising because all of the plants received the same amount of water but different potassium amounts.

TR estimation using machine learning is not yet an established practice. However, the ability to create different models for the different parts of the day was used to develop different TR models that took advantage of the high temporal and spectral resolution database along with the corresponding PA attributes. The best performing model was for the afternoon, similar to our previous findings using a different approach [28]. Whereas the previous results were obtained by comparing TR variance between parts of the day, and the correlation to spectral bands to capture those differences, this work used a more general approach to capture and model the differences. While both studies were conducted on a single plant species in one custom greenhouse setup, both found that the afternoon is more suitable for TR estimation. Nonetheless, this contradicts the common assumption that around noon, when solar radiation is strongest and TR is highest, is the best time for remote sensing of plant physiological attributes [43,44,45,46]. This raises a question as to the optimal time to conduct remote or direct spectral data acquisition for vegetation monitoring. Moreover, TR estimations of plants that received different salinity treatments showed that with a generic trained model, salinity affects the estimations. As salinity increased, the model’s performance decreased, and there was more variance in the results. The implication of these results for field conditions, and more specifically for remote sensing, is meaningful. Namely, high-salinity soils may introduce variance into the measurements, making field estimations of TR more prone to errors. In addition, TR estimation of plants that received different potassium treatment revealed that the prediction of a generic trained model is most affected by potassium deficit and performs best when potassium is at a normal levels for the crop.

The most accurate model (82% accuracy) for classifying low-potassium-treated samples was confined to the low and high potassium treatments. The effect of medium levels of potassium in relation to spectral changes was less intense than surplus levels of potassium. The two models to classify low and medium and low and high potassium levels were more sensitive to potassium deficit. When all of the treatments were combined, the overall accuracy decreased. The spectral characteristics of high levels of potassium proved to be more significant. This is evident from the model’s greater sensitivity and precision for high levels of potassium. These different models provide more insight into the effect of potassium on the spectra and, in turn, on the models’ sensitivity to alterations in potassium levels. However, in a real greenhouse or even in field scenarios, practical considerations regarding yield result in surplus addition of potassium to crops. The ability to isolate plants with low potassium levels is important, especially at the beginning of the growing season, which is the growth period covered in this experiment. This ability is an important addition to the grower’s decision-making toolbox, facilitating the targeting of specific areas or specific plants that suffer from potassium shortage to optimize yield.

The advantages of feature extraction and bands selection to reduce the dimensionality of the data and exclude none contributing spectral channels has supported the increased interest in multispectral sensors in space, airborne and field domains [47,48]. However, it was shown that a small subset of bands is not sufficient to achieve the same classification accuracy as obtained in hyperspectral domains. Similarly, Pimstein et al. [18] reported the necessity of many spectral bands to estimate potassium in wheat. While the high spectral resolution was key to identifying the most important spectral bands for this task, the high temporal resolution was more important in achieving high accuracy. In addition, the comparison with a traditional algorithm (SVM) showed a clear advantage for the XGBoost algorithm in terms of accuracy. Nonetheless, the SVM has only two tunable hyperparameters, which makes it much faster to optimize and train.

This research proved reliable for potassium estimation in pepper plants, and additional research using different plants under different growing conditions is warranted. In addition, the ability to create inclusive datasets such as the one suggested here and estimating TR may help future breeding programs identify bad hybrids and focus on successful ones.

## 5. Conclusions

High temporal hyperspectral measurements using a proximal hyperspectral camera along with a functional phenotyping system proved to be reliable as a nondestructive method for the difficult tasks of potassium-treatment classification and TR estimation. The best statistics were achieved for the classification of low and high levels of potassium, mimicking a common agronomic scenario of surplus fertilization and local shortages. Moreover, the best time to estimate TR using the custom physiological-hyperspectral system in the greenhouse was counterintuitively found to be the afternoon—after the TR has peaked at around noon and is declining. In addition, high salinity levels resulted in a decrease in TR estimation. Creating a large dataset favored the task at hand from both an agronomic and machine-learning algorithm point of view. The high spectral resolution provided by the hyperspectral technology was essential to achieving high accuracy. To the best of our knowledge, the current study is the first to provide in-vivo results without performing destructive analyses of plant tissue. Future studies should expand other plants, treatments, and field scenarios to create additional databases that will support practical agronomic decision-making.

## Figures and Tables

**Table 1:** Number of samples in the database for the different analyses.

	Classification		Transpiration-Rate Estimation					
		Count	Period	Count	Salinity	Count	Potassium	Count
	Low K	1644	Morning	2376	H_2_O	1611	Low	1061
	Medium K	1795	Noon	1970	Medium	1355	Medium	849
	High K	1422	Afternoon	3271	High	1345	High	1325
Sum		4861 ^a^		7617		4311		3199

^a^ Classification of potassium (K) treatments was carried out for one level of salinity (control treatment). Transpiration rate estimation was carried out on the entire dataset, and salinity effects were tested on the salinity treatments experimental table.

**Table 2:** Selected hyperparameter values for each model.

Parameters		Values Tested	Selected Value per Model				
			Classification			Regression	
			Low–Medium	Low–High	Low–Medium–High	Spectra	Spectra & Ambient
XGBoost	Number of trees	100, 300, 800, 1000	800	1000	1000	1000	1000
	Eta (learning rate)	0.1, 0.01	0.1	0.1	0.1	0.01	0.01
	Tree depth	2, 4, 6, 8, 10, 12	12	8	8	8	8
	*L*1	0.00001, 0.01, 0, 0.1, 1, 100	0.00001	0.00001	0.00001	0	0.01
	*L*2	0.00001, 0.01, 0, 0.1, 1, 100	0.00001	0.01	0.00001	0	0.1
	Percent of features	0.3, 0.5, 0.8	0.5	0.5	0.5	0.3	0.3
SVM	*C*	0.1, 1, 10, 100	100	100	100		
	*γ*	1, 0.1, 0.01, 0.001	1	1	1		

**Table 3:** XGBoost Classification results. (best values at each classification are in bold)

	All Bands			Best Bands		
Classified treatments	Accuracy	Sensitivity	Precision	Accuracy	Sensitivity	Precision
Low	80%	**83%**	**81%**	68%	**71%**	**70%**
Medium		78%	79%		66%	66%
Low	**83%**	**86%**	80%	70%	**75%**	70%
High		80%	**86%**		65%	70%
Low	79%	76%	73%	**77%**	**79%**	**81%**
Medium		80%	81%		75%	78%
High		**81%**	**83%**		76%	72%

## Data Availability

Not applicable.

## References

[1] Dalal A., Bourstein R., Haish N., Shenhar I., Wallach R., Moshelion M. (2019). Dynamic Physiological Phenotyping of Drought-Stressed Pepper Plants Treated With “Productivity-Enhancing” and “Survivability-Enhancing” Biostimulants. Front. Plant Sci..

[2] Inoue Y. (1991). Remote and Real-Time Sensing of Transpiration and Stomata Resistance Based on Infrared Thermometry. Jarq Ibaraki.

[3] Gosa S.C., Lupo Y., Moshelion M. (2019). Quantitative and comparative analysis of whole-plant performance for functional physiological traits phenotyping: New tools to support pre-breeding and plant stress physiology studies. Plant Sci..

[4] Sade N., Gebretsadik M., Seligmann R., Schwartz A., Wallach R., Moshelion M. (2009). The Role of Tobacco Aquaporin1 in Improving Water Use Efficiency, Hydraulic Conductivity, and Yield Production Under Salt Stress. Plant Physiol..

[5] Jarolmasjed S., Sankaran S., Kalcsits L., Khot L.R. (2018). Proximal hyperspectral sensing of stomatal conductance to monitor the efficacy of exogenous abscisic acid applications in apple trees. Crop. Prot..

[6] Rozenstein O., Haymann N., Kaplan G., Tanny J. (2019). Validation of the cotton crop coefficient estimation model based on Sentinel-2 imagery and eddy covariance measurements. Agric. Water Manag..

[7] Negin B., Moshelion M. (2017). The advantages of functional phenotyping in pre-field screening for drought-tolerant crops. Funct. Plant Biol..

[8] Rapaport T., Hochberg U., Shoshany M., Karnieli A., Rachmilevitch S. (2015). ISPRS Journal of Photogrammetry and Remote Sensing Combining leaf physiology, hyperspectral imaging and partial least squares-regression (PLS-R) for grapevine water status assessment. ISPRS J. Photogramm. Remote Sens..

[9] York L.M. (2018). Functional phenomics: An emerging field integrating high-throughput phenotyping, physiology, and bioinformatics. J. Exp. Bot..

[10] Furbank R.T., Tester M.A. (2011). Phenomics—technologies to relieve the phenotyping bottleneck. Trends Plant Sci..

[11] Pieruschka R., Poorter H. (2012). Phenotyping plants: Genes, phenes and machines. Funct. Plant Biol..

[12] Zhao C., Zhang Y., Du J., Guo X., Wen W., Gu S., Wang J., Fan J. (2019). Crop Phenomics: Current Status and Perspectives. Front. Plant Sci..

[13] Nagler P.L., Glenn E.P., Lewis Thompson T., Huete A. (2004). Leaf area index and normalized difference vegetation index as predictors of canopy characteristics and light interception by riparian species on the Lower Colorado River. Agric. For. Meteorol..

[14] Bannari A., Morin D., Bonn F., Huete A.R. (1995). A review of vegetation indices. Remote Sens. Rev..

[15] Glenn E.P., Nagler P., Huete A. (2010). Vegetation Index Methods for Estimating Evapotranspiration by Remote Sensing. Surv. Geophys..

[16] Mahajan G.R., Sahoo R., Pandey R.N., Gupta V.K., Kumar D. (2014). Using hyperspectral remote sensing techniques to monitor nitrogen, phosphorus, sulphur and potassium in wheat (*Triticum aestivum* L.). Precis. Agric..

[17] Pandey P., Ge Y., Stoerger V., Schnable J.C. (2017). High Throughput In vivo Analysis of Plant Leaf Chemical Properties Using Hyperspectral Imaging. Front. Plant Sci..

[18] Pimstein A., Karnieli A., Bansal S.K., Bonfil D.J. (2011). Exploring remotely sensed technologies for monitoring wheat potassium and phosphorus using field spectroscopy. Field Crops Res..

[19] Rozenstein O., Paz-Kagan T., Salbach C., Karnieli A. (2014). Comparing the Effect of Preprocessing Transformations on Methods of Land-Use Classification Derived from Spectral Soil Measurements. IEEE J. Sel. Top. Appl. Earth Obs. Remote Sens..

[20] Zhong L., Hu L., Zhou H. (2019). Deep learning based multi-temporal crop classification. Remote Sens. Environ..

[21] Chen T., Guestrin C. XGBoost: A scalable tree boosting system. Proceedings of the ACM SIGKDD International Conference on Knowledge Discovery and Data Mining, Association for Computing Machinery.

[22] Georganos S., Grippa T., VanHuysse S., Lennert M., Shimoni M., Wolff E. (2018). Very High Resolution Object-Based Land Use–Land Cover Urban Classification Using Extreme Gradient Boosting. IEEE Geosci. Remote Sens. Lett..

[23] Abdi A.M. (2020). Land cover and land use classification performance of machine learning algorithms in a boreal landscape using Sentinel-2 data. Gisci. Remote Sens..

[24] Sandino J., Pegg G.S., Gonzalez F., Smith G.R. (2018). Aerial Mapping of Forests Affected by Pathogens Using UAVs, Hyperspectral Sensors, and Artificial Intelligence. Sensors.

[25] Loggenberg K., Strever A., Greyling B., Poona N. (2018). Modelling Water Stress in a Shiraz Vineyard Using Hyperspectral Imaging and Machine Learning. Remote Sens..

[26] Kawamura K., Mackay A.D., Tuohy M.P., Betteridge K., Sanches I.D., Inoue Y. (2011). Potential for spectral indices to remotely sense phosphorus and potassium content of legume-based pasture as a means of assessing soil phosphorus and potassium fertility status. Int. J. Remote Sens..

[27] Colombo R., Meroni M., Marchesi A., Busetto L., Rossini M., Giardino C., Panigada C. (2008). Estimation of leaf and canopy water content in poplar plantations by means of hyperspectral indices and inverse modeling. Remote Sens. Environ..

[28] Weksler S., Rozenstein O., Haish N., Moshelion M., Wallach R., Ben-Dor E. (2020). A Hyperspectral-Physiological Phenomics System: Measuring Diurnal Transpiration Rates and Diurnal Reflectance. Remote Sens..

[29] Halperin O., Gebremedhin A., Wallach R., Moshelion M. (2017). High-throughput physiological phenotyping and screening system for the characterization of plant-environment interactions. Plant J..

[30] Cakmak I. (2005). The role of potassium in alleviating detrimental effects of abiotic stresses in plants. J. Plant. Nutr. Soil Sci..

[31] Zhang X., Liu F., He Y., Gong X. (2013). Detecting macronutrients content and distribution in oilseed rape leaves based on hyperspectral imaging. Biosyst. Eng..

[32] Otsu N. (1979). A Tlreshold Selection Method from Gray-Level Histograms. IEEE Trans. Syst. Man Cybern..

[33] Willmott C.J., Robeson S.M., Matsuura K. (2012). A refined index of model performance. Int. J. Clim..

[34] Fauvel M., Chanussot J., Benediktsson J.A. Evaluation of Kernels for Multiclass Classification of Hyperspectral Remote Sensing Data. Proceedings of the 2006 IEEE International Conference on Acoustics Speed and Signal Processing Proceedings.

[35] Melgani F., Bruzzone L. (2004). Classification of Hyperspectral Remote Sensing. IEEE Trans. Geosci. Remote Sens..

[36] Pedregosa F., Varoquaux G., Gramfort A., Michel V., Thirion B., Grisel O., Blondel M., Prettenhofer P., Weiss R., Dubourg V. (2011). Scikit-learn: Machine Learning in Python. J. Mach. Learn. Res..

[37] Marschner H., Marschner P. (2012). Marschner’s Mineral Nutrition of Higher Plants.

[38] Gitelson A.A., Merzlyak M.N. (2003). Relationships between leaf chlorophyll content and spectral reflectance and algorithms for non-destructive chlorophyll assessment in higher plant leaves. J. Plant Physiol..

[39] Curran P.J. (1989). Remote sensing of foliar chemistry. Remote Sens. Environ..

[40] Gamon A., Peuelas J., Field C.B. (1992). A Narrow-Waveband Spectral Index That Tracks Diurnal Changes in Photosynthetic Efficiency. Remote Sens. Environ..

[41] Zhao D., Reddy K.R., Kakani V.G., Reddy V. (2005). Nitrogen deficiency effects on plant growth, leaf photosynthesis, and hyperspectral reflectance properties of sorghum. Eur. J. Agron..

[42] Penuelas J.P.J., Pinol J., Ogaya R., Filella I. (1997). Estimation of plant water concentration by the reflectance Water Index WI (R900/R970). Int. J. Remote Sens..

[43] Zhou J., Chen H., Zhou J., Fu X., Ye H., Nguyen H.T. (2018). Development of an automated phenotyping platform for quantifying soybean dynamic responses to salinity stress in greenhouse environment. Comput. Electron. Agric..

[44] Liu S., Peng Y., Du W., Le Y., Li L. (2015). Remote Estimation of Leaf and Canopy Water Content in Winter Wheat with Different Vertical Distribution of Water-Related Properties. Remote Sens..

[45] Gamon J.A., Field C.B., Bilger W., Fredeen A.L. (1990). Remote sensing of the xanthophyll cycle and chlorophyll fluorescence in sunflower leaves and canopies. Oecologia.

[46] Gerhards M., Schlerf M., Rascher U., Udelhoven T., Juszczak R., Alberti G., Miglietta F., Inoue Y. (2018). Analysis of Airborne Optical and Thermal Imagery for Detection of Water Stress Symptoms. Remote Sens..

[47] Svensgaard J., Roitsch T., Christensen S. (2014). Development of a Mobile Multispectral Imaging Platform for Precise Field Phenotyping. Agronomy.

[48] Lurbe C.B., Brinkhoff J., Quayle W.C., Hornbuckle J. (2019). Monitoring the Effects of Water Stress in Cotton using the Green Red Vegetation Index and Red Edge Ratio. Remote Sens..

